# Effects of non-vascularized adipose tissue transplantation on its genetic profile

**DOI:** 10.1080/21623945.2021.1889815

**Published:** 2021-03-01

**Authors:** Jeannine S. Schreiter, L. O Kurow, S Langer, M Steinert, L Massier

**Affiliations:** a Klinik am Rosental GmbH Leipzig, Leipzig, Germany; b Department of Orthopedics, Traumatology and Plastic Surgery; c Department of Thoracic Surgery, University Hospital Leipzig, Leipzig, Germany; University Hospital Leipzig, Leipzig, Germany; e Medical Department III – Endocrinology, Nephrology, Rheumatology, University of Leipzig, Leipzig, Germany

**Keywords:** Adipose tissue, fat transplantation, gene expression, insulin resistance, gene ontology

## Abstract

Subcutaneous adipose tissue (SAT) is recognized as a highly active metabolic and inflammatory tissue. Interestingly, adipose tissue transplantation is widely performed in plastic surgery via lipofilling, yet little is known about the gene alteration of adipocytes after transplantation. We performed an RNA-expression analysis of fat transplants before and after fat transplantation.

In C57BL/6 N mice SAT was autologously transplanted. Samples of SAT were analysed before transplantation, 7, and 15 days after transplantation and gene expression profiles were measured.

Analysis revealed that lipid metabolism-related genes were downregulated while inflammatory and extracellular matrix related genes were up-regulated 7 and 15 days after transplantation. When comparing gene expression profile 7 days after transplantation to 15 days after transplantation developmental pathways showed most changes.

## Introduction

Subcutaneous white adipose tissue (SWAT) has been shown to be an important player in the development of the metabolic syndrome, as it is a highly active metabolic tissue and is involved in inflammatory processes [1]. Its different cell components such as adipocytes, preadipocytes, macrophages, and other cells summarized as the stromal vascular fraction [2], act together as an endocrine organ with a huge inflammatory capacity [3]. Adipocytes and preadipocytes express themselves mRNA of many cytokines and chemokines and are therefore not only able to interfere in inflammation, via interaction and activation of the adipose tissue macrophages [4,5], but can also directly act as inflammatory tissue[5].

In plastic surgery precisely this SWAT is used for free fat transplantation. Interestingly, most studies on fat transplantation address the viability of transplanted fat tissue and its ability to fulfill the requirements of volume augmentation. Opposing to this huge amount of studies, to our knowledge, no study has addressed the impact of transplantation itself on the metabolic and other biological functions of the transplanted tissue itself. We know that transplanted fat tissue can ameliorate skin texture when transferred to scar tissue[6] or reduce fibrosis in radiated tissue[7]. Hence, this tissue is biologically active after transplantation. But we ignore the effect the transplantational stress has on the biological functions of the fat tissue. As gene expression changes are the first answers to external physiological demands of the cell, we decided to assess the genetic response of the adipose tissue after transplantation.

To address this question we undertook a study, where we used autologous fat transplantation in a murine dorsal skinfold chamber model and performed gene expression analysis before transplantation, 7, and 15 days after transplantation. The model of the dorsal skinfold chamber was chosen on the one hand, to allow for best identification of the transplanted fat tissue after 7 and 15 days. On the other hand, as the graft size is rather small, particle size correlates with that reached in lipofilling[8], using the dorsal skinfold chamber allowed to clearly identify the graft after 7 and 15 days after transplantation. Our data were completed by haematoxylin and eosin staining and immunohistochemical analysis of three selected antibodies corresponding to significantly differentially expressed genes.

## Material

### Animals

We used a total of 22 female C57BL/6 N mice, with an average age of all animals of 11.7 (±1.4) weeks, mean weight was 21.8 (±1.4)g. SWAT specimen of 4 animals were used before transplantation for RNA analysis. All remaining animals (N = 18) had an autologous fat transplantation into a dorsal skinfold chamber as described previously[9]. Mean fat transplant weight was 0.01 (±0.01)g. Randomized 9 animals were euthanized via cervical dislocation 7 days after transplantation and tissue samples were used for RNA analysis. The remaining 9 animals were euthanized 15 days after transplantation, and transplanted adipose tissue was collected for RNA-analysis.

We monitored Body weight and temperature of mice at days 0, 3, 7, 10, and 15 after transplantation. We did not see significant changes in Body weight and temperature during our experiments.

The weight of transplanted tissue was about 0.8 mg while average body weight was 21.9 g, hence, the metabolic changes within the transplanted tissue itself do not significantly influence the total metabolic profile of the animals in this experimental setting.

The study was approved by the local governmental animal care committee (protocol number 01/16) and conducted in accordance with the German legislation on protection of animal and the National Institute of Health Guidelines for the Care and Use of Laboratory Animals. It was run at the Medical Experimental Centre of the University of Leipzig. All mice were housed in pathogen-free facilities at 22 ± 2°C on a 12-h light/dark cycle. Animals were bred and kept in the animal laboratories at the University of Leipzig, and were fed a standard chow diet. Animals had ad libitum access to water at all times. No animal dyed during the experiments. We decided to work with female animals, because of the significantly higher percentage of women having lipofilling done compared to male patients in the human clinical setting and the gender-specific differences of adipose tissue [10,11].

### RNA extraction

Fat tissue was extracted from the dorsal skinfold chamber before transplantation and from euthanized mice 7 and 15 days after transplantation. They were fresh frozen and stored at −80°C. Total RNA was extracted from the tissue samples, following the manufacturer’s protocol. Before microarray analysis was done, RNA integrity and concentration were examined on an Agilent Fragment Analyser (Agilent Technologies, Palo Alto, CA, USA) using the HS RNA Kit (Agilent Technologies) according to the manufacturer’s instructions.

Samples had an RNA integrity number of 8.68 (± 0.47) on average and a 28s/18s ratio of 1.66 (± 0.11). One sample of the group 7 days after transplantation had to be withdrawn because of a poor RIN of 2.2.

### Microarray analysis

Microarray analysis was conducted at the Core Unit DNA Technologies (Core Facilities of the Faculty of Medicine; University of Leipzig). cRNA was prepared from 100 ng of total RNA hybridized to GeneChip Clariom S arrays (ThermoFisher Scientific) according to the manufacturer’s instructions. The arrays were scanned with a third-generation Affymetrix GeneChipScanner 3000.

### Data analysis

Affymetrix GeneChip data were extracted from fluorescence intensities and were scaled in order to normalize data for inter-array comparison using Transcriptome Analysis Console (TAC) 4.0.2 software according to instruction of the manufacturer (ThermoFisher Scientific).

### Transcriptome analyses

Gene expression profiles were measured using Clariom S mouse arrays (ThermoFisher Scientific, Waltham, MA, USA) in the Core Unit DNA Technologies of the University Leipzig Medical Faculty according to the manufacturers’ instructions. Data were background corrected and normalized by applying Robust Multi array Averaging (RMA) using the Affymetrix Expression Control™ software (ThermoFisher Scientific). Subsequent analyses were performed in R v3.6.1^12^ using *limma* v3.40.6^13^ and employing a linear fit model and Empirical Bayes statistics for differential expression.

Pathways analyses were performed in GSEA v4.0.3^14^ with standard parameters, 1000 phenotype permutations and using KEGG pathways database as reference[15]. Additionally, gene ontology analyses were performed using the web-based GOrilla platform[16]. Multiple testing problems were addressed by employing Benjamini-Hochberg correction[17] and a q value below 0.05 was considered significant in all cases. All figures were made using *ggplot2* v3.3.0^18^ or *VennDiagram* v.1.6.20[19].

### Histology of fat grafts

At days 0, 7, and 15 the mice were euthanized and fat graft specimens were placed in zinc-formaldehyde and used for histological and immunofluorescence examination. Afterwards they were either embedded in paraffin or in gelatine. Sections of 7 µm thickness were cut and stained. We used the following primary antibody for immunofluorescence: Anti-MS2 antibody ab255608 (abcam, Berlin, Germany), Anti-Osteopontin antibody ab8448 (abcam), and Fatty Acid Synthase (C20G5) Rabbit mAb (Cell Signalling Technology, Frankfurt a. M., Germany). As conjugated secondary antibody, we used Alexa Fluor 555 (both Cell Signalling Technology).

## Results

**Figure 1.**
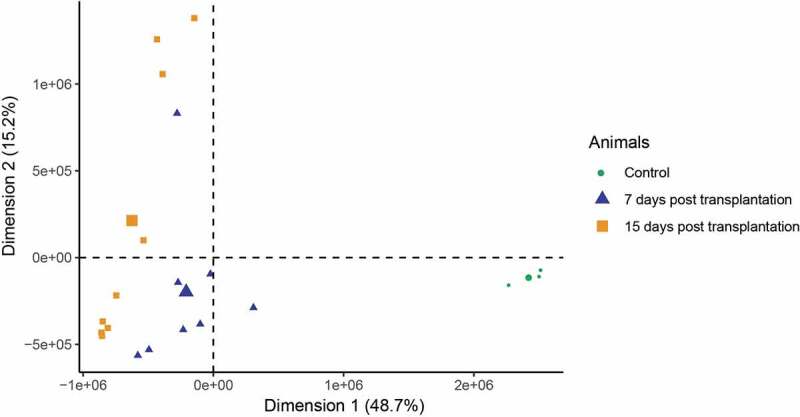
Principal component analysis (PCA) of gene expression profiles of C57Bl/6 N subcutaneous white adipose tissue before (red), 7 (green) and 15 days (blue) after non-vascularized autologous transplantation (page 4)

Hierarchical clustering of gene expression profiles for each sample in a PCA revealed a gene expression shift after transplantation. Indeed, both gene expression profiles after 7 and 15 days after transplantation differed similarly to the basal expression profile before transplantation (Figure 1).

### Differentially expressed genes (DEGs) in response to time after transplantation

The Clariom S mouse array was used to profile RNA expressions for the adipose tissue sample directly after explantation and after 7 and 15 days after transplantation (N = 4, 8, and 9, respectively). We compared the differentially expressed genes (DEGs) at day 0 to day 7 (0vs7) and day 0 to day 15 (0vs15) to assess the impact of time after transplantation.

**Figure 2.**
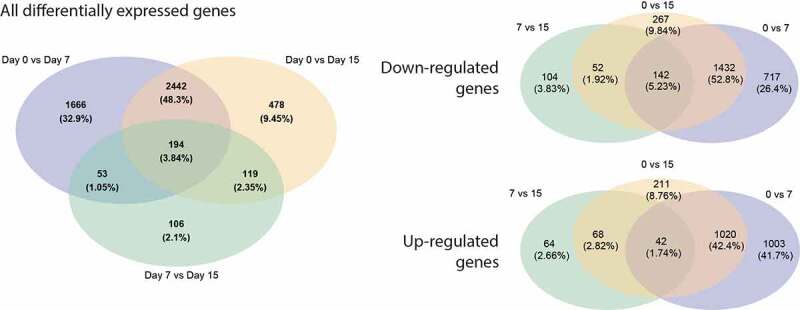
VENN diagram for the overlapping genes of the different groups representing the number of all genes (a) that were upregulated (b) or downregulated (c) after transplantation. The intersection represents genes that were regulated in both or all conditions. FDR <0.05 (page 5)

Out of 21,903 detected genes, a total of 4355 genes were differentially expressed 7 days after transplantation and 3233 genes were differentially expressed 15 days after transplantation (FDR <0.05). A total of 2291 genes and 1893 genes were downregulated and 2065 and 1341 were upregulated after 7 and 15 days after transplantation, respectively (Figure 2).

We analysed the overlapping DEGs that had FDR-q < 0.05 in all three comparison groups (0vs7, 0vs15, and 7vs15) and visualized them in a Venn diagram (Figure 2). 1.74% of all upregulated DEGs showed a significant upregulation when comparing 0vs7, 0vs15, and 7vs15, supposing that time after transplantation had a cumulative effect on their expression. Likewise, 5.32% of all downregulated DEGs showed a significant decrease in every time comparison.

**Figure 3.**
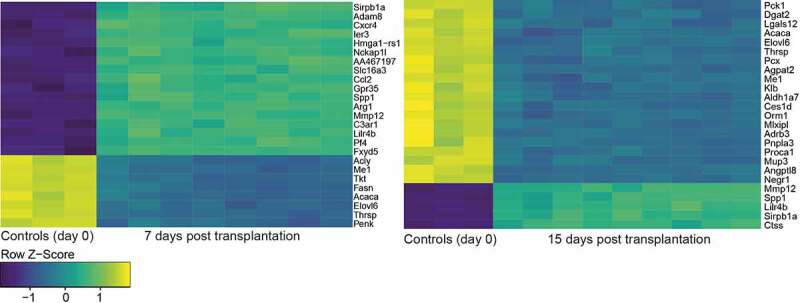
Heatmaps of the 25 most significantly differentially expressed genes in adipose tissue before and 7 days (a) and 15 days after transplantation (b) (page 5)

**Figure 4.**
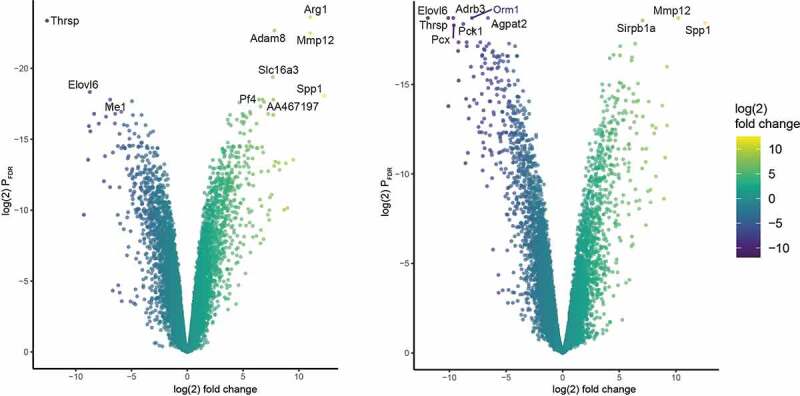
Volcano plots of all differentially expressed genes in adipose tissue before and 7 (a) and 15 days (b) after transplantation (page 5)

**Figure 5.**
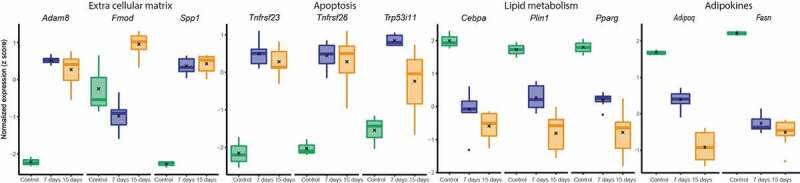
Box plot selected gene expression in adipose tissue before and 7 (a) and 15 days (b) after transplantation (page 5) referring to extra cellular matrix, apoptosis, lipid metabolism and adipokines

The most significantly differentially expressed genes between the different groups were identified using volcano plot and heatmap filtering (Figures 3 and 4). Seven days and 15 days after transplantation volcano plots and heat maps identified the genes Sirpb1a, Adam8, Spp1, and Mmp12 being the most significantly differentially upregulated genes and the genes Me1, Acaca, Elovl6, and Thrsp being most significantly downregulated when comparing both groups after transplantation to the group of the non-transplanted tissue (Figure 3, 4, and 5).

Going in line with our hypothesis, that the fat graft shows an early remodelling process after transplantation, we found that the most significantly downregulated DEGs were energy metabolism-related genes, while the upregulated DEGs were linked to extracellular matrix (ECM) remodelling and inflammation.

### Gene expression of genes related to extracellular matrix remodelling and inflammation are upregulated after transplantation

Sirpb1a, Spp1, and Mmp12 were most significantly upregulated in both groups. Sirpb1a encodes for Signal-regulatory protein beta 1, which is a transmembrane receptor, that acts as a phagocytic receptor on macrophages[20]. It is involved in inflammatory response[21]. Secreted phosphoprotein 1 (SPP1) gene encodes for the protein Osteopontin, which is a phosphorylated protein of the ECM, that has a dominant role in B and T cell control[22], in cancer development and is a gene relevant for ECM[23]. It is highly expressed in adipose tissue macrophages[24]. MMP-12 belongs to the group of matrix metalloproteinase and is with MMP-2 and MMP-9 responsible for elastolysis as it occurs during wound healing and inflammatory diseases[25]. (Figure 5).

### Gene expression of energy metabolism-related genes is downregulated after transplantation

The genes Me1, Acaca, Elovl6, and Thrsp were most significantly downregulated in both groups and are all responsible for energy metabolism. Malic enzyme 1 (ME1) is involved in the glycolytic and the citric acid cycles and plays an important role for NADPH production, glutamine metabolism, and lipogenesis[26]. Acaca gene encodes for Acetyl-CoA carboxylase which catalyzes the ATP-dependent carboxylation of actetyl-CoA and is therefore a very important regulator in fatty acid biosynthesis[27]. Elovl6 encodes for the elongation of long-chain fatty acids family member 6. Equally to the former proteins, Elovl6 is involved in fatty acid biosynthesis as it catalyzes the elongation of saturated and monounsaturated fatty acids[28]. Thyroid hormone responsive protein (Thrsp) is associated to lipogenesis. A *Thrsp* Null Mouse (Thrsp^*tm*^1^*cnm*^) model was linked to an improved glucose tolerance[29]. (Figure 5).

### Gene ontology

**Figure 6.**
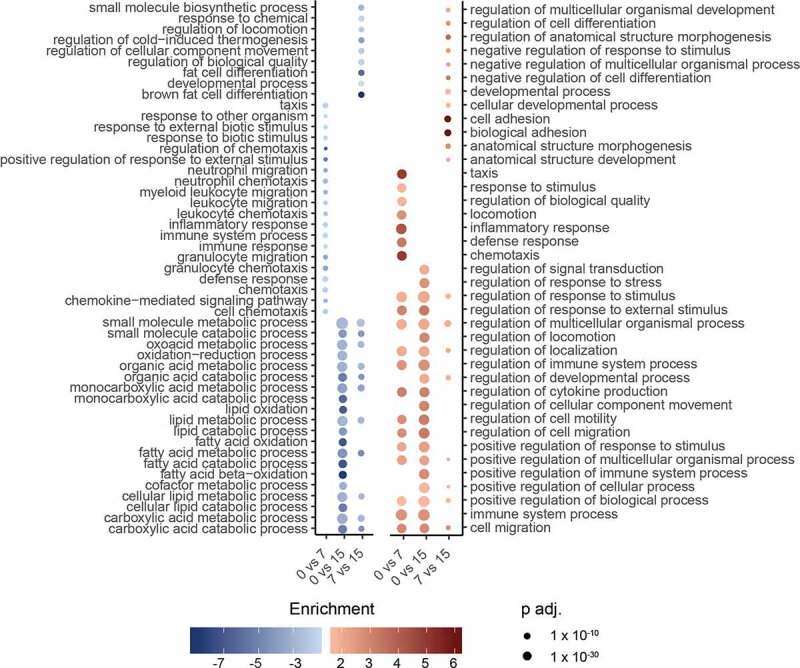
Bubble plot of gene ontology analysis using GOrilla tool (biological process) of differentially expressed pathways of all downregulated pathways (blue) and all upregulated pathways (red). All with FDR <0.01, before transplantation, 7, and 15 days after transplantation (page 6)

**Figure 7.**
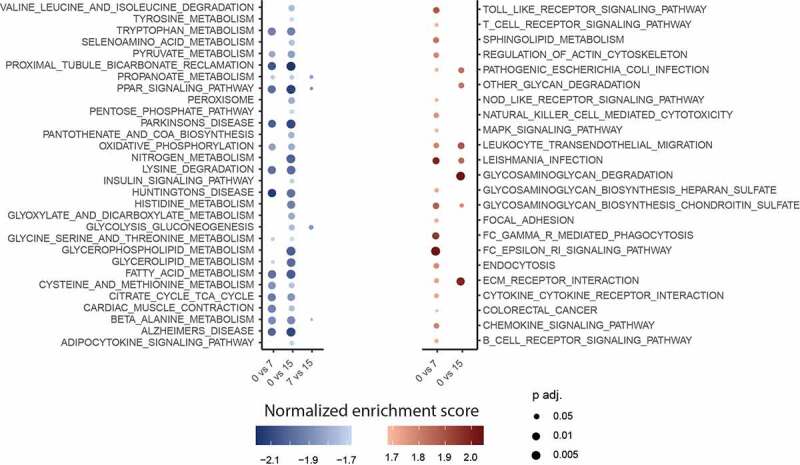
Bubble plot of gene ontology analysis using GSEA and KEGG database (biological process) of the top 20 downregulated pathways (a) and top 20 upregulated pathways (b). All FDR <0.01. using GSEA, before transplantation, 7, and 15 days after transplantation (page 6)

Using the GOrilla tool [30], we analysed the biological processes linked to the DEGs. We confirmed that most downregulated processes were involved in metabolic processes, while the upregulated processes corresponded to inflammatory and remodelling processes (Figure 6). Pathway analysis done with GSEA using KEGG database verified the repartition of the biological up- and downregulated pathways (Figure 7). Interestingly, downregulation of metabolic involved pathways seems to start rather 7 to 15 days after transplantation and is not an early genetic response to transplantation. Indeed, when comparing the genetic pathway profiles before and 7 days after transplantation, changes in inflammatory and remodelling processes dominate changes in metabolic involved pathways.

Remarkably, when comparing DEGs and gene ontology changes between 7 and 15 days after transplantation, analysis showed most changes in genes and gene pathways linked to cell differentiation and developmental processes (Figure 5 A and B). As these pathways only showed up when comparing day 7 to day 15, we assume, that it is a phenomenon that is initiated only after several days of transplantation, unlike the upregulation of immune response associated pathways which started right after transplantation. These different patterns of pathway regulation reflect the dynamic answer of the transplanted tissue according to the time after transplantation.

Through volcano plot and heat map filtering, we identified the most significantly differentially regulated genes when comparing the adipose tissue gene profile 7 and 15 days after transplantation (Figure 7).

### Mup-family genes are significantly downregulated 15 days after transplantation

**Figure 8.**
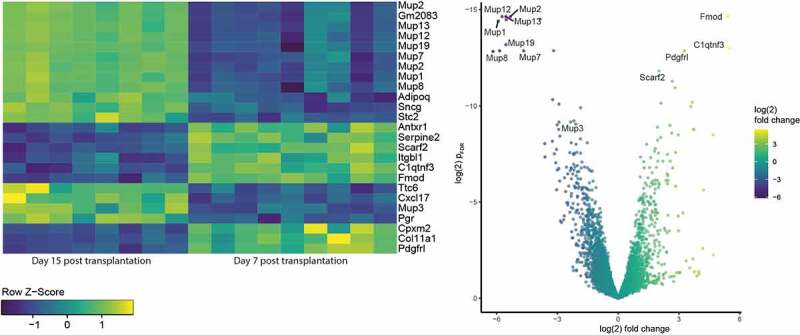
Heatmap (a) and volcano plot (b) of the differentially expressed genes in adipose tissue 7 and 15 days after transplantation, highlighting the mup-family (page 6)

Most interestingly, several genes of the Major urinary protein (Mup) family were significantly downregulated when comparing gene expression after 7 and after 15 days after transplantation (Figure 7): Mup1, Mup-2, Mup3, Mup 5, Mup 7, Mup 8, Mup 12, Mup13, Mup 19, and Mup 20 had adjusted p-values ranging from p = 0.4x10^−6^ to 0.0054 (Figure 8).

Mups are proteins mostly found in rodents’ urine that a transcribed in a sex-dependent way. Literature describes behavioural and pheromone effects of these proteins. Moreover, they have been associated to energy metabolism and glucose homoeostasis in rodents. In humans close functional or sequence homologs have not been found. Members of the lipocalin family in humans however, are closely related to the mup in mice[31].

### Fibromodullin expression is significantly increased one week after transplantation

Fmod gene expression is most significantly upregulated when comparing 7 to 15 days after transplantation (p_adj_ 4,3x 10^−^[7]). Fmod encodes for fibromodullin, which is a known potent angiogenesis stimulator[32]. Gene ontology reveals that they are mostly involved in cell adhesion, morphological and developmental processes.

### Histological and immunohistochemical dies

To validate our genetic evaluation, we performed HE-stains and IHC for MS-2, Osteopontin, and Fatty Acid Synthase. We chose these proteins, as their encoding genes Adam8, Spp1, and Fasn showed significant changes of gene expression after transplantation according to our study and corresponding antibodies in mice were available.

**Figure 9.**
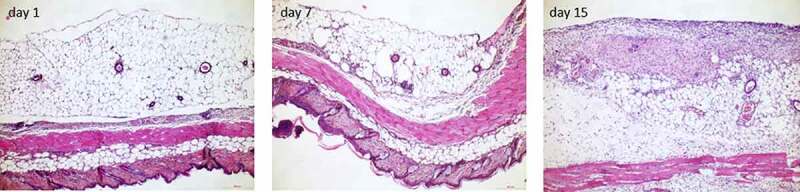
HE stain of the transplanted fat tissue within the dorsal skinfold chamber 1, 7 and 15 days after transplantation. An increase of fibrotic changes with oedema in the surroundings of the fat transplant is observable. Scale bar 200 µm each (page 7)

HE stain of the transplanted fat tissue within the dorsal skinfold chamber 1, 7 and 15 days after transplantation showed an increase of fibrotic changes with oedema in the surroundings of the fat transplant (Figure 9).

**Figure 10.**
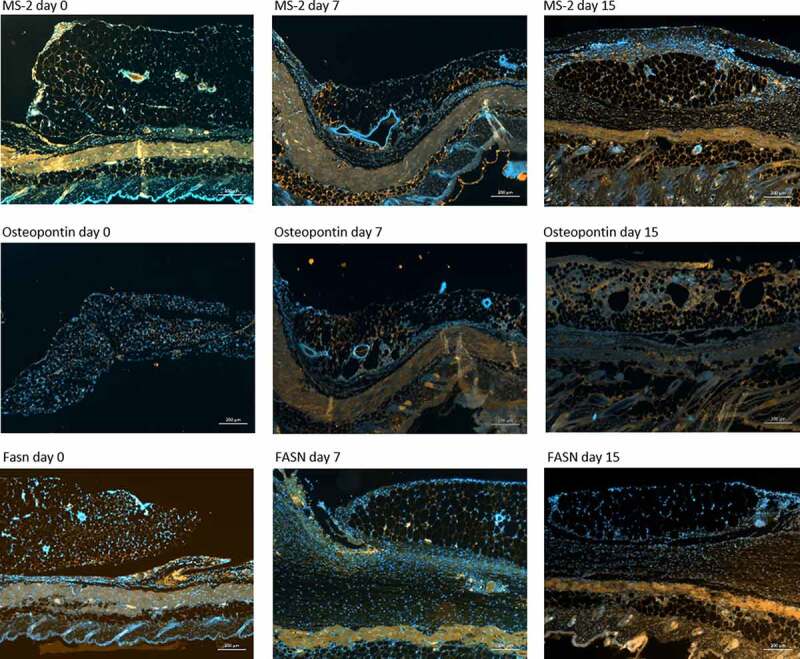
Immunohistochemical dies of transplanted adipose tissue at the day of transplantation, 7 and 15 days after transplantation. Blue: DAPI dye of the nuclei. First row: Orange: MS-2 staining within the fat graft in the dorsal skinfold chamber. Second row orange: Osteopontin positive cells. Third row: orange. FASN positive cells. Scale bar 200 µm each (page 7)

For MS-2, Osteopontin, and Fatty Acid Synthase we saw confirming trends going in line with our previous genetic findings: On the one hand, we found an increase in MS-2 (gene Adam8) and Osteopontin (Spp1) with time after transplantation within the transplanted fat tissue (Figure 10 first and second row). On the other hand, consistent with the downregulation of the gene expression of Fasn, we saw a decrease of fatty acid synthase positive cells with increasing time after transplantation (Figure 10, third row).

Due to relatively small sample sizes of our histology experiments, we can only describe trends.

## Discussion

Adipose tissue is a very attractive tissue in plastic surgery used for remodelling, as it is easily manipulated and gained. Yet, adipose tissue is not an inert tissue but indeed a potent endocrine organ that can actively trigger inflammation[33]. Many studies underline the injurious effects adipose tissue has in obesity regarding the development of the metabolic syndrome. Adipose tissue has a tremendous modulatory power as it secretes diverse cytokines and triggers inflammation. During fat transplantation, this tissue is not only mechanically manipulated but also imposed to the transplantational stress. Moreover, in some cases even its microenvironment changes when lipofilling is performed into a muscle[34]. Adipose-derived stem cells have been shown to improve the graft volume retention in lipofilling, and it is supposed to happen through secretion of growth factors by ADSCs[35]. Considering the adipose tissue capacity to secrete cytokines, react on micro environmental changes and interfere in inflammation, we aimed to analyse the effect of transplantation on the transplanted adipose tissue.

As changes in the gene expression are the first detectable changes when answering external demands, we decided to compare gene expression in non-transplanted subcutaneous tissue to gene expression profiles 7 and 15 days after transplantation using microarray analysis. So far, no study has examined the effect of transplantation on the genetic expression profile of the transplanted adipose tissue. Lindegren et al. analysed changes of gene expression of irradiated breast tissue after lipofilling treatment[36]. Garza et al. evaluated single-cell gene expression after fat transplantation[37]. However, Zuk and co-workers highlighted the multicellular composition of the adipose tissue [2,28]. Therefore, in our opinion, it is most interesting to analyse the effect of transplantation on the complete grafted tissue as a whole. Moreover, analysing the complete fat tissue avoids a collagenase isolation procedure which has been shown to provoke itself marked alterations in gene expression[38].

We undertook a murine transplantational study using the dorsal skinfold chamber model. We saw, that there is a more severe change in gene expression profiles when comparing fat tissues gene expression before and after transplantation, than when comparing the gene expression profiles 7 days to 15 days after transplantation. This underlines the high impact transplantation has on the gene expression answer. Most changes were seen in the field of extracellular matrix and inflammation involved genes, being significantly upregulated, while energy metabolism linked gene and pathways were mostly downregulated. These findings substantiate the remodelling and inflammatory potential of adipose tissue and mirror genetic changes that have been described obese white adipose tissue showing an increased expression of genes encoding for ECM components [39,40]. Hence, there might be a similar development in obese white adipose tissue and transplanted lean adipose tissue. Studies revealed that an increased activation of ECM goes along with an increased adipocyte death due to a restricted space within the tissue. What conclusions can be set for the fate of transplanted adipose tissue, if it shows similar remodelling events than obese white adipose tissue? With an increase of ECM and inflammation encoding genes the constitution of the transplanted tissue would further shift to a fibrotic tissue, losing adipocytes. “Previous histologic examination with haematoxylin and eosin staining of transplanted fat tissue revealed increasing fibrosis within the fat transplant [41–44] and a decrease of the adipocytes’ size and shape [45]. We verified these changes in HE staining of samples of transplanted adipose tissue within the dorsal skinfold 1, 7, and 15 days after transplantation supporting our observation regarding the up-regulation of ECM and fibrosis-related genes (Figure 9).

It is unlikely that the injurious effects of obese white adipose tissue such as increasing glucose intolerance will arise from constricted transplanted adipose tissue in a systemic relevant extent, as the amount of the transplanted fat tissue is marginal compared to the total body fat mass. Though it is worth knowing that transplanted adipose tissue shows a comparable genetic profile than inflammatory obese white adipose tissue. Immunohistochemical staining for selected antibodies (FASN, Osteopontin and MS-2) confirmed the results from the genetic profiling (Figure 10).

Remarkably, we observed a highly significant downregulation of major urinary proteins when comparing genes expression profiles 7 to 15 days after transplantation. Literature to these proteins is rather scarce, presumably because these proteins do not have direct homolog in humans. Mups have been described as pheromones. This here presented work may rise attention to possible other significance of these proteins. Zhou et al. suggested that Mup1 plays a role in the systemic glucose and lipid metabolism regulation in hepatocytes[46]. And a differential expression rate of MUP-1 was described in subcutaneous white adipose tissue compared to epididymal fat tissue in mice[38]. SWAT showed higher MUP-1 gene expression and MUP-1 transcript levels than epididymal fat tissue.

Going in line with the idea of a turnover from mature dying adipocytes to preadipocytes within transplanted tissue [47–49], some of the most downregulated genes found in this study such as Adiponectin, FASN, and Mup are genes typically expressed in adipocytes and not in preadipocytes[38].^,4^ C1qtnf3, instead, which was found to be upregulated when comparing gene expression 7 and 15 days after transplantation, is expressed in 4 days old adipocytes[50]. This strengths the theory, that there is a turn-over from dying mature adipocytes in the grafted tissue towards more extracellular matrix components and more preadipocytes[51]. The downregulation of genes involved in metabolism going aside an increase of inflammatory processes may be a hint for the death of the adipocytes. Interestingly gene pathways involved in fat cell differentiation were equally down- and upregulated when comparing gene ontology profile 7 to 15 days after transplantation. Further analysis of downstream pathways of these processes will clarify the fate and the activity of the transplanted adipose tissue.

## Conclusion

Lipofilling is a commonly used technique in plastic surgery as the tissue is easily gained and manipulated. Though, adipose tissue is nonetheless a highly active endocrine organ, capable of interfering in inflammation. Gene expression profile of lean adipose tissue changes under the transplantational stress, mimicking that of obese white adipose tissue. Though the load of transplanted tissue compared to the total amount of fat tissue is neglectable regarding systemic metabolic changes, local effect still needs to be elucidated.
